# Optimization of 3D-Visualization of Micro-Anatomical Structures of the Human Inner Ear in Osmium Tetroxide Contrast Enhanced Micro-CT Scans

**DOI:** 10.3389/fnana.2018.00041

**Published:** 2018-05-22

**Authors:** Thomas van den Boogert, Marc van Hoof, Stephan Handschuh, Rudolf Glueckert, Nils Guinand, Jean-Philippe Guyot, Herman Kingma, Angelica Perez-Fornos, Bart Seppen, Lejo Johnson Chacko, Anneliese Schrott-Fischer, Raymond van de Berg

**Affiliations:** ^1^Division of Balance Disorders, Department of Otorhinolaryngology, Head, and Neck Surgery, Faculty of Health Medicine and Life Sciences, School for Mental Health and Neuroscience, Maastricht University Medical Centre, Maastricht, Netherlands; ^2^VetCore Facility for Research, University of Veterinary Medicine Vienna, Vienna, Austria; ^3^Department of Otorhinolaryngology, Medical University Innsbruck, Innsbruck, Austria; ^4^Department of Clinical Neurosciences, Service of Otorhinolaryngology, Head, and Neck Surgery, Geneva University Hospitals, Geneva, Switzerland; ^5^Vestibular Laboratory, Faculty of Physics, Tomsk State National Research University, Tomsk, Russia

**Keywords:** contrast enhancement staining, image processing, Micro-CT, temporal bones, 3D rendering, inner ear, cochlear implants, vestibular implants

## Abstract

**Introduction:** Knowledge of the neuro-anatomical architecture of the inner ear contributes to the improvement and development of cochlear and vestibular implants. The present knowledge is mainly based on two-dimensional images (histology) or derived models that simplify the complexity of this architecture. This study investigated the feasibility of visualizing relevant neuro-anatomical structures of the inner ear in a dynamic three-dimensional reproduction, using a combination of staining, micro-CT imaging and an image processing algorithm.

**Methods:** Four fresh cadaveric temporal bones were postfixed with osmium tetroxide (OsO_4_) and decalcified with EDTA. Micro-CT was used for scanning at 10 μm (4 scans) and 5.5 μm (1 scan) voxel resolution. A new image processing algorithm was developed and the scans were visualized in open source software.

**Results:** OsO_4_ enhanced the contrast in all scans and the visualization was substantially improved by the image processing algorithm. The three-dimensional renderings provided detailed visualization of the whole inner ear. Details were visible up to the size of individual neurons, nerve crossings and the specific neuro-anatomical structures such as the tunnel of Corti.

**Conclusion:** The combination of OsO_4_, micro-CT and the proposed image processing algorithm provides an accurate and detailed visualization of the three-dimensional micro-anatomy of the human inner ear.

## Introduction

When impaired, the functions of malfunctioning sensory structures in the peripheral vestibular and auditory system can be replaced by neural prosthetics. Their function is to substitute the sensory modality of balance (van de Berg et al., 2011) and hearing (Eisen, 2009), by stimulating nerves with the electrical equivalent of healthy stimuli. Cochlear implants (CI) are able to assist recipients in restoring the ability to communicate to near-normal levels (Raman et al., 2011; Nicholas and Geers, 2013). However, they do not achieve the same acuity of hearing as age-matched healthy controls and mostly leave recipients unable to enjoy the complex traits of music (Drennan, 2008). One factor of importance might be that the design of these neural implants does not take into account the intricate three dimensional (3D) neural architecture in the labyrinth (Lindemann, 1969). It can be hypothesized that an improved ability to image the micro-anatomy of the inner ear would increase the performance of such an implant, by contributing to improvements in electrode design and processing strategies. The same arguments hold true for the newly developed vestibular implant (VI) (Marianelli et al., 2012).

Many computational models used for the improvement of electrode design and processing strategies represent “averages” and “assumptions” of the human cochlear anatomy (Kalkman et al., 2016), taking into account, on a functional level, the place-pitch map for humans (Vermeire et al., 2015). Other conventional anatomical models make use of extensive manual segmentation, represented in a mathematical or finite element model (Vogel, 1999; Poznyakovskiy et al., 2008; Bradshaw et al., 2010; Braun et al., 2012). These models are based on computed tomography (CT) or histology and rely on a high level of human interpretation, such as segmentation. The individual variability in the 3D neural folding pattern in the cochlea remains unknown and is difficult to investigate in detail using techniques in use today. To allow studying these individual detailed patterns of human anatomy, a 3D model is needed, based on volumetric renderings to minimize human interpretation. To increase identifiability of the neural structures, involved in inner ear implantology, osmium tetroxide (OsO_4_) stain is used to selectively increase the contrast of unsaturated fatty acids in cell membranes and lipid rich structures in myelinated neural tissue (Metscher, 2009). Associated noise is to be removed for two- and three dimensional (2D and 3D) visualization.

The objective of this study was to develop a protocol for image processing after staining on post-mortem human labyrinths to visualize the sensory structures of the inner ear with a high level of contrast in 3D, with a minimum of human interpretation and manual segmentation. It was hypothesized that a 3D reconstruction of the inner ear would be able to demonstrate the neural pathways, the location of the sensory organs and their inter-structural relationships in 3D.

## Materials and methods

### Ethics

The bodies were donated to the Division of Clinical and Functional Anatomy of the Innsbruck Medical University by subjects who had given their informed consent prior to death, specifically for the use of their bodies for scientific and educational purposes (McHanwell et al., 2012; Riederer et al., 2012). All specimens were anonymized. This study was in accordance with the Declaration of Helsinki (amended version 2013). No review of an ethical board was required. This study was in accordance with local legislation.

### Inclusion, preparation, staining, and scanning of temporal bones

Four temporal bone samples were harvested and included for staining from adult and senior Caucasians without known inner ear or outer ear pathology (Table 1). Specimens were preserved and stained at the department of anatomy, where after micro-CT image acquisition was performed. The paper of Glueckert et al., (submitted) described in detail, the preparation, staining and scanning of the same set of included temporal bones. Scanning and reconstruction parameters are displayed in Table 2. All four samples were scanned in 10 μm resolution. The cochlear partition of sample S1 was also scanned in 5.5 μm resolution.

**Table 1:** Demographics.

**Sample**	**Age at death**	**Gender**	**Hearing impairment**	**Ethnicity**
S1	85 years	Male	No	Caucasian
S2	29 years	Male	No	Caucasian
S3	85 years	Male	No	Caucasian
S4	50 years	Female	No	Caucasian

*The age of the samples varied between 29 and 85 years. Three samples were masculine, one feminine. All specimens were Caucasian and were without recorded hearing impairment*.

**Table 2:** CT-scan parameters.

**Sample**	**Voxel size (μm)**	**Bit depth**	**Dimensions**	**Size (GB)**
S1	10/5.5	16 Bit	1,002 × 1,322 × 1,763/897 × 1,378 × 1,701	3.92/3.91
S2	10	16 Bit	1,255 × 1,248 × 1,500	4.37
S3	10	16 Bit	1,146 × 992 × 1,780	3.77
S4	10	16 Bit	1,624 × 1,000 × 1,100	3.32

*All samples were scanned in 10 μm resolution. The cochlear partition of sample S1 was also scanned in 5.5 μm resolution. Volume dimensions were adjusted to match the dimensions of the inner ear*.

### Image processing and qualitative assessment

The 3D image volumes were processed by image processing filters available in Wolfram Mathematica (Version 10.4; Wolfram Research, Inc., Champaign, Il, USA). Two authors (TvdB and MvH) qualitatively assessed the outcomes in consensus. Images were visually assessed for noise, contrast and sharpness of detail. When an outcome was considered an improvement, it was used in a successive step of image processing. Analysis was performed on a workstation [3,5-GHz 6-core 12 MB L3-cache, RAM - 64 GB (4x 16 GB DDR3 ECC), 512 GB PCIe-SSD, dual AMD FirePro D500 GPU's, 3 GB GDDR5 VRAM]. Version 4.5.0-1 of 3D slicer (2015) (Pieper et al., 2004; Fedorov et al., 2012) and version 2.0.0 of Fiji (2015) (Schindelin et al., 2012; Rasband, 2015) were used for additional manual image segmentation and computer file format conversion.

#### Noise reduction algorithm for 2D visualization

Volumes were imported into Mathematica (Table 3, step 1). Histograms of all slices were matched to a single chosen slice (reference image) to equalize contrast in the Z-axis of the volume (Table 3, step 2). Subsequently, volumes were filtered in 12 iterations by two different nonlinear local filters (Table 3, step 3, 4, 5) used for edge preserving smoothing. Finally, volumes were sharpened (Table 3, step 6).

**Table 3:** Noise reduction algorithm for 2D visualization.

**Noise reduction step of algorithm 2D**	**Function**	**Settings**	**Timing (s)**
1	Image loading	−	9
2	Histogram transform	reference image	648
3	Guided filter	*r* = 1, ε = 1	810
4	Bilateral filter	σ = 1, μ = 0.00017	22,212
5	Guided filter	*r* = 1, ε = 1	810
6	Sharpen	–	969

*A single chosen slide functions as rreference image, to equalize contrast in the Z axis. Total processing time for sample S1 is 25,438 s. Function names correspond to function names in Wolfram Mathematica*.

#### Noise reduction algorithm for 3D visualization

**Figure 1:**
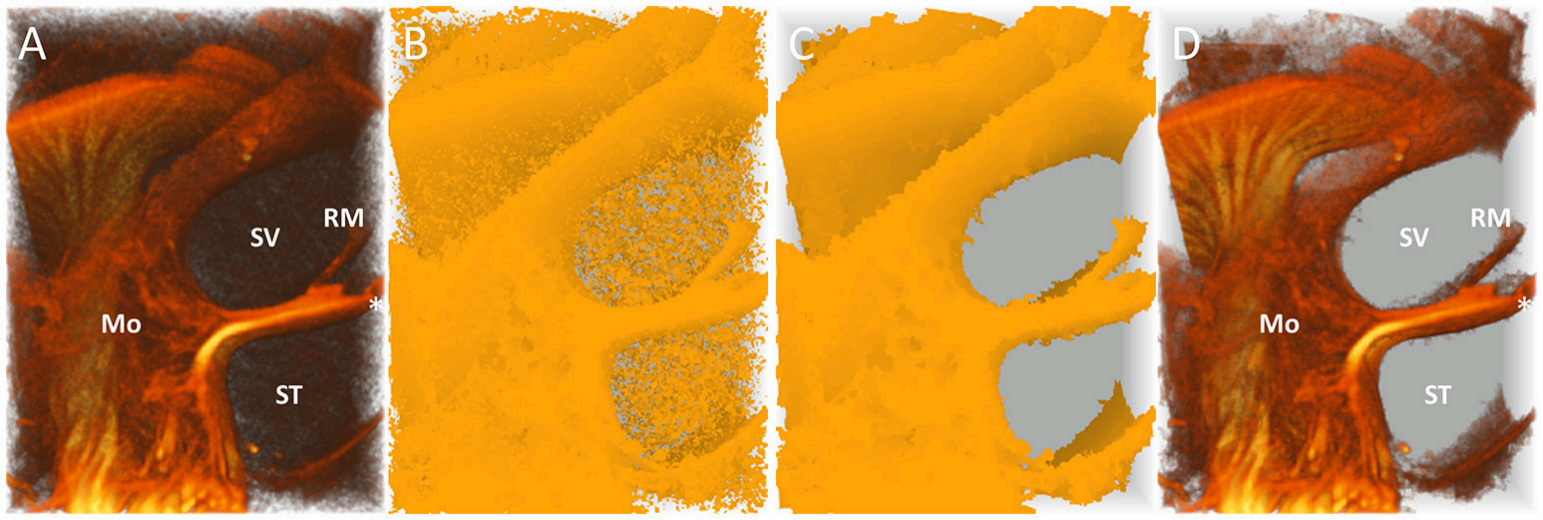
Automatic segmentation and noise reduction algorithm for 3D visualization. 3D renderings of a part of the cochlea in sample S1 (5.5 μm resolution) showing the steps of automatic segmentation and noise reduction for 3D visualization corresponding to results in Figure 2. **(A)** Pre-processed volume (Table 3–Step 3) with structures of interest obscured by unconnected random noise. **(B)** Displays creating a mask (Table 4–Step 4). **(C)** Small unconnected particles are removed from the mask (Table 3–Step 5) and the mask is dilated (Table 3–Step 6,7). **(D)** The resulting volume after masking (Table 3–Step 8). ^*^Tunnel of Corti. ST, Scala tympani; SV, Scala vestibule; RM, Reissner's membrane; Mo, Modiolus.

Volumes were imported into Mathematica (Table 4, step 1) and filtered by a nonlinear local filter, used for edge preserving smoothing (Table 4, step 2). Bright objects, up to a specified radius, were extracted from the volume using a tophat transform (Table 4, step 3). The obtained processed volumes (Figure 1A) were used to construct binary masks (Table 4, step 4) (Figure 1B). Since small unconnected objects resembled noise in the volumes, they were removed from the mask using “GeodesicOpening” (Table 4, step 5). To ensure all desired structures were enclosed in the residual mask, it was padded using a morphological closing (Table 4, step 6). Subsequently it was dilated with one pixel in every dimension using a cross-matrix (Table 4, step 7) (Figure 1C). The binary mask (Figure 1C) was used to segment the processed volume (Figure 1A; Table 4, step 8), as illustrated in Figure 1D. Volumes were manually segmented, to exclude circumjacent structures.

**Table 4:** Noise reduction algorithm for 3D visualization.

**Noise reduction step of algorithm 3D**	**Function**	**Settings**	**Timing (s)**
1	Image loading	–	9
2	Guided filter	*r* = 1, ε = 1	810
3	Tophat transform	Crossmatrix, *r* = 64	7,284
4	Binarize	0.035	1
5	Geodesic opening	15	393
6	Closing	2	305
7	Dilation	Crossmatrix, *r* = 1	92
8	Image multiply	–	6

*Total processing time for sample S1 is 8,900 s. Function names correspond to function names in Wolfram Mathematica. Image multiply, multiplies the mask with the resulted volume obtained after Tophat transform*.

### Volume rendering

Mathematica and 3D slicer were used for volume rendering. The scans were rendered using the VTK ray casting technique (Pieper et al., 2004; Schroeder et al., 2006). Color and transparency functions were chosen subjectively to optimize image quality and the ability to distinguish separate anatomical structures.

## Results

### Performance of contrast enhanced staining and spatial resolution

**Figure 2:**
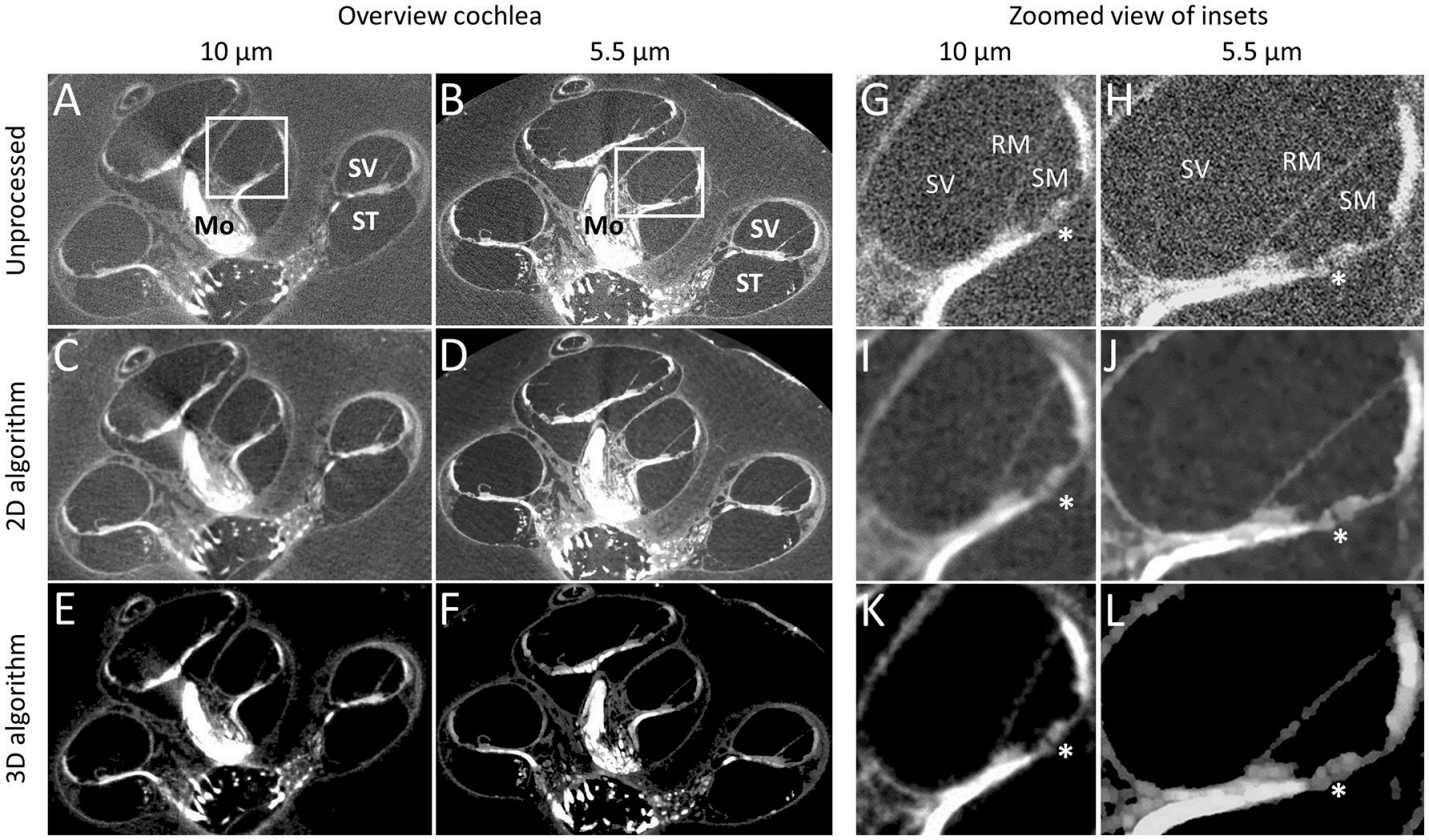
Performance of contrast enhancement staining and noise reduction algorithms. Graphs show the cochlea in sample S1 in 10 and 5.5 μm resolution before processing **(A,B,G,H)**, after processing for 2D visualization **(C,D,I,J)** and 3D visualization **(E,F,K,L)**. Tunnel of Corti depicts the approximate smallest resolvable feature (28 μm) after noise reduction for 2D and 3D visualization. ^*^Tunnel of Corti. ST, Scala tympani; SV, Scala vestibule; RM, Reissner's membrane; Mo, Modiolus.

The OsO_4_ stain provided strong contrast in all included, unprocessed, micro-CT scans (Figures 2A,B,G,H). Myelinated nerve tissue could be clearly distinguished (Figures 2A,B). A magnified part of the cochlea (Figures 2G,H) demonstrates the level of detail and the difference obtained between a 10 μm and 5.5 μm resolution. A small substructure, in this case the tunnel of Corti which measures approximately 28 μm in diameter illustrates the increased resolving power for the 5.5 μm voxel resolution scan (Figures 2G,H).

#### Qualitative assessment of the performance of noise reduction algorithms

For the 2D algorithm, noise in the volumes was visually decreased by the nonlinear local filters used (Figures 2C,D,I,J), compared to the original volumes (Figures 2A,B,G,H). Sharpening visually reduced the effect of blurring due to the smoothing in the noise reduction process. The algorithm for 3D visualization removed noise and increased contrast (Figures 2E,F,K,L), further than the 2D algorithm while reducing some detail (Figures 2I,J vs. Figures 2K,L). A higher local contrast and sharp delineation was achieved for the 3D algorithm. The automatic segmentation process was then able to segment the designated anatomical features achieving the result in Figure 1D. It was estimated that manual segmentation took 30 min.

### Three-dimensional visualization of the inner ear anatomy

**Figure 3:**
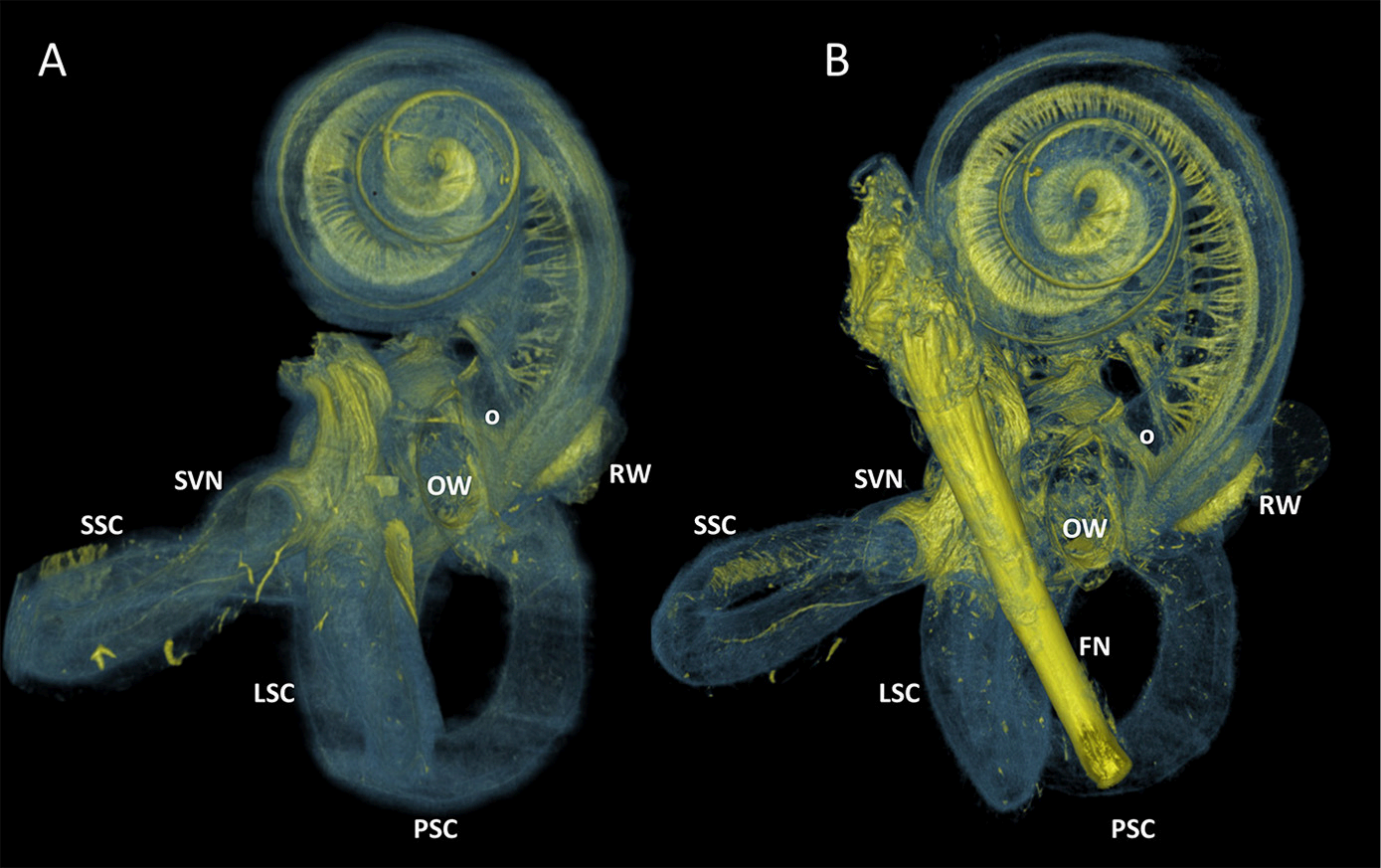
3D renderings of a sample before and after processing with the 3D algorithm. Color rendering (sample S1) based on intensities to distinguish nervous tissue (yellow), bone and membranous structures (blue). **(A)** Manual segmented volume without post-processing. Facial nerve was not segmented. **(B)** Volume with 3D algorithm applied. FN, Facial nerve; OW, Oval window; RW, Round window; SVN, Superior branch of the vestibular nerve; SSC, LSC, PSC Superior, lateral and posterior semicircular canal respectively. _O_ Neural structure, indicative for vestibule-cochlear anastomosis of Oort.

**Figure 4:**
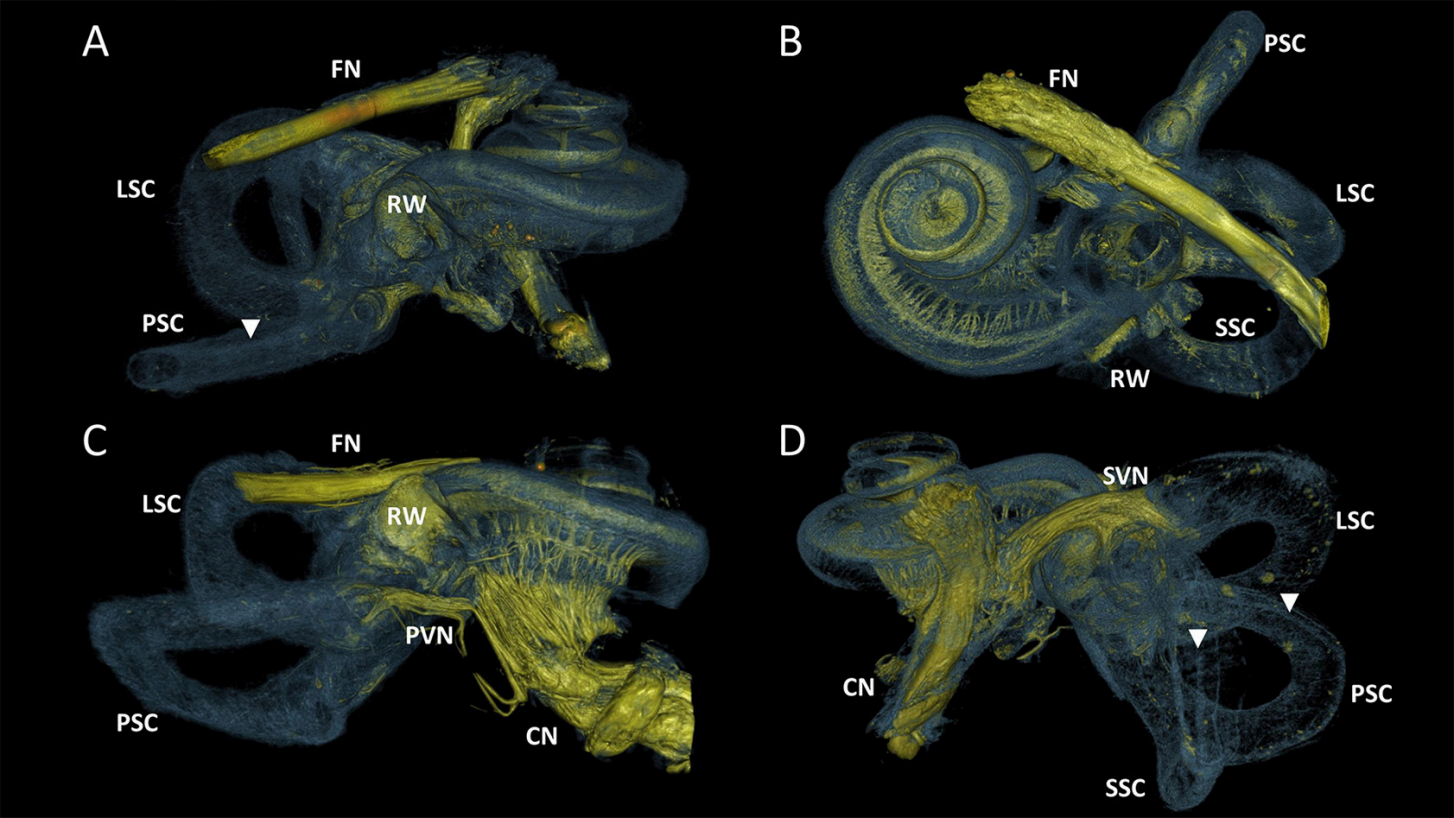
3D renderings of the entire labyrinth of the processed samples from different perspectives. Color rendering based on intensities to distinguish nervous tissue (yellow), bone and membranous structures (blue). **(A)** Sample S1. Cochlear nerve (CN) was removed during dissection. **(B)** Sample S2. Radially and spirally oriented fibers within the cochlea are visible. **(C)** Sample S3. Posterior branch of the vestibular nerve (PVN) connects the cupula of the posterior semicircular canal (PSC) with the CN. **(D)** Sample S4. Superior branch of the vestibular nerve (SVN) connect the cupula of the lateral and superior semicircular canal (LSC and SSC) to the CN. FN, Facial nerve; RW, Round window. Membranous labyrinth could be discerned from its bony encasement (▾).

Figure 3 visualized the qualitative improvement of contrast and sharper delineation in a comparison between volume rendering results of the same sample without any form of post-processing (Figure 3A) and with the 3D algorithm applied (Figure 3B). After post-processing the four samples, all of the 10μm resolution 3D renderings had sufficient spatial resolution to visualize the entire inner ear with appurtenant neural tissue and sensory organs (Figure 4). Visual tracing of the trajectory of myelinated nerve fibers in the cochlear nerve and vestibular nerve branches (superior and posterior) was possible. In some parts, the membranous labyrinth could be discerned from its bony encasement (▾ in Figures 4, **6**)

**Figure 5:**
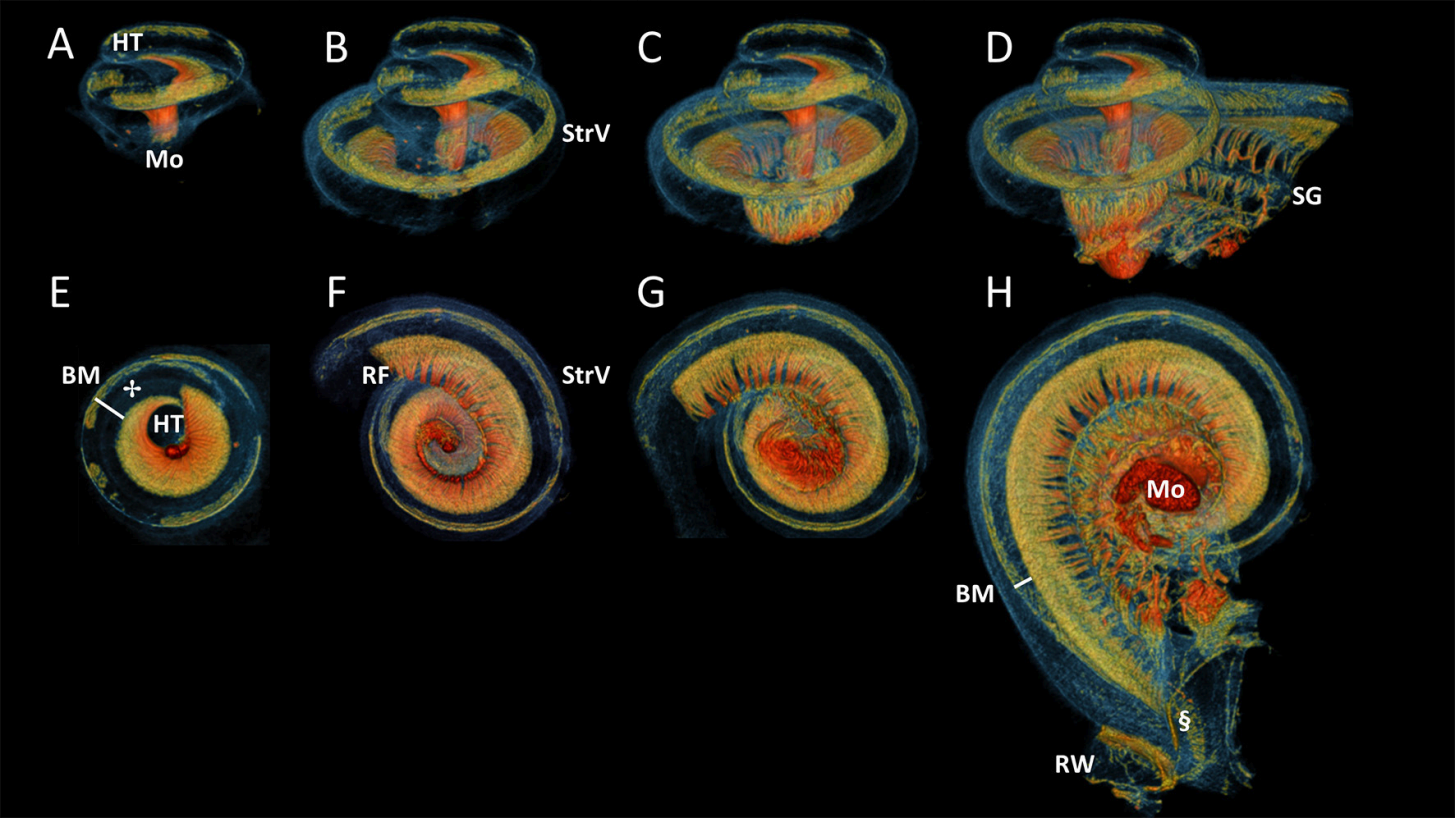
Side and bottom view of progressive virtual sections of the cochlea. Color renderings S4, side view: **(A–D)**, bottom view: **(E–H)** based on intensities to distinguish nervous tissue (orange and red), bone and membranous structures (blue) and stria vascularis (StrV) (green). These show the spirally and radially branching neural innervation of the cochlea from apex, the helicotrema (HT), to base, the round window (RW). The radial fibers **(**RF**)** fan out from the modiolus (Mo) through Rosenthal's canal, where the spiral ganglion (SG) cells are located. The RF connect to hair cells on organ of Corti (✢).The Basilar membrane (BM) widens apically, compared to the width at the base of the cochlea.

Figure 5 displayed successive virtual sections of the cochlea, seen from the side (A-D) and from apex toward base (E-H). This revealed the spiral folding of myelinated radial nerves toward the centrally located spiral ganglion, connecting the hair cells (✢ in Figure 5E) in the most apical part of the organ of Corti (not shown), near the helicotrema. The basally radial fibers branched out of the peripherally located spiral ganglion and were directed outward to connect to hair cells on the organ of Corti. These myelinated radially oriented nerve fibers were not traceable after losing their myelin at the habenula perforata. The difference in width of the basilar membrane (approximated from nerve fibers to the stria vascularis) could be seen to differ in the apex (Figure 5E) in comparison to the base (Figure 5H).

**Figure 6:**
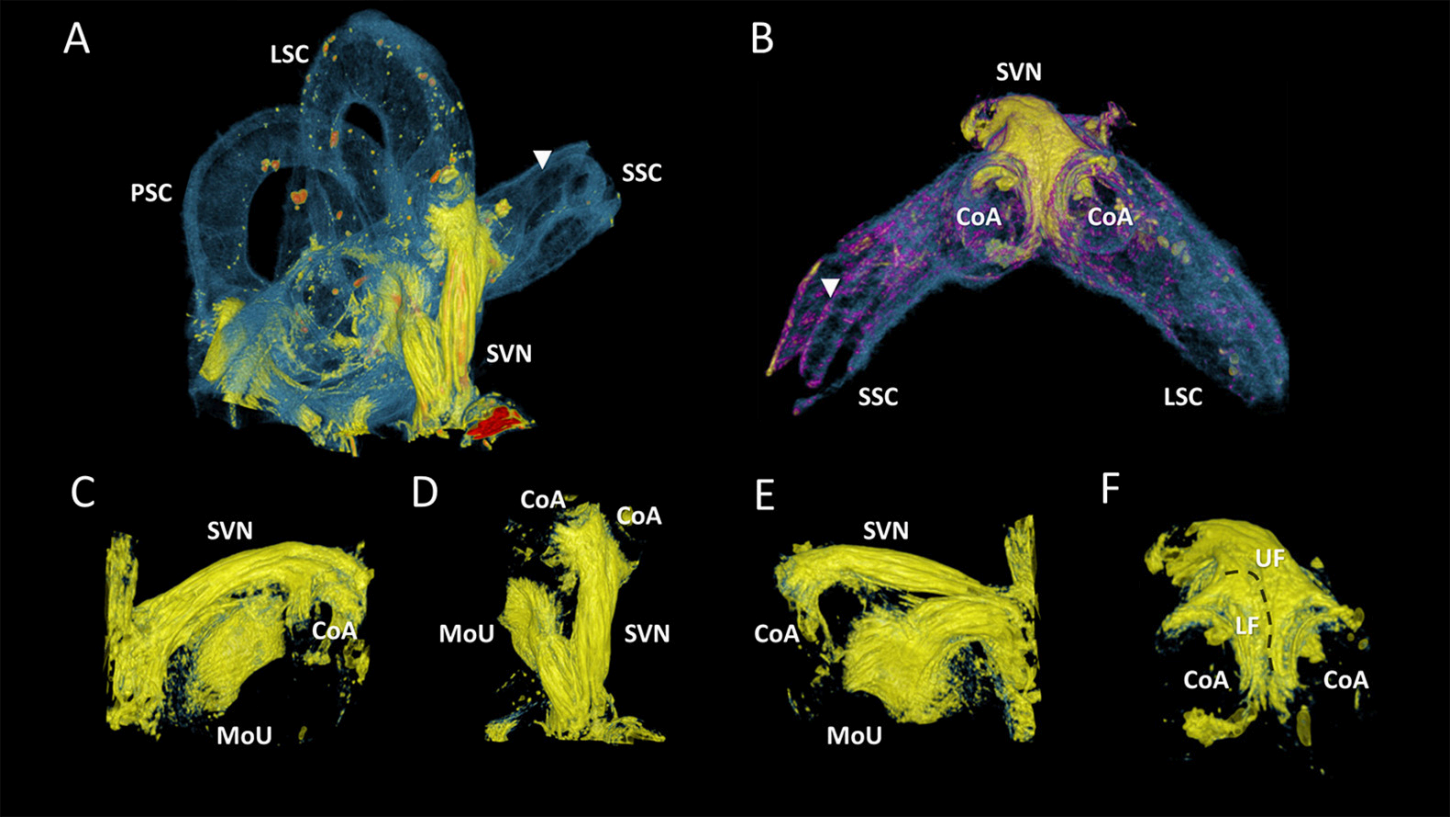
Close-ups of the superior branch of the vestibular nerve. Color renderings (sample S2) based on intensities to distinguish nervous tissue (yellow), bone and membranous structures (blue and purple). **(A)** shows the posterior, lateral and superior semicircular canal (PSC, LSC, SSC) and the superior branch of the vestibular nerve (SVN). **(B)** Close-up of the SVN, connecting to the cristae of the ampulla (CoA) of the SSC and LSC. **(C)** Frontal view of SVN terminating in the macula of the utricle (MoU). **(D)** Same viewpoint as **(A)**, with neural structures extracted. **(E)** Back view of the MoU. **(F)** shows the SVN from the same viewpoint as **(B)**. Nerve fibers from the cristae differentiate in an upper and lower bundle (UF, LF) for respectively the LSC and SSC Membranous labyrinth could be discerned from its bony encasement (▾).

Figure 6 showed close-ups of the superior branch of the vestibular nerve that ended in the utricle and in the ampullae of the superior and lateral semi-circular canals. The nerve endings in the ampullae of the semi-circular canals formed a crescent shape (Figures 6A,B,D,F), and connected to the cristae (not visible). These two nerve fiber bundles folded over each other with a lower and upper fiber bundle (Figure 6F). The superior branch of the vestibular nerve bended toward the macula of the utricle (Figures 6A,D), before terminating in a roughly round plaque of nerve fibers in the utricle (Figures 6C,E). The neuro-epithelial side of this plaque (looked upon in Figure 6C) was hollow in the middle, but convexly shaped toward its contour.

**Figure 7:**
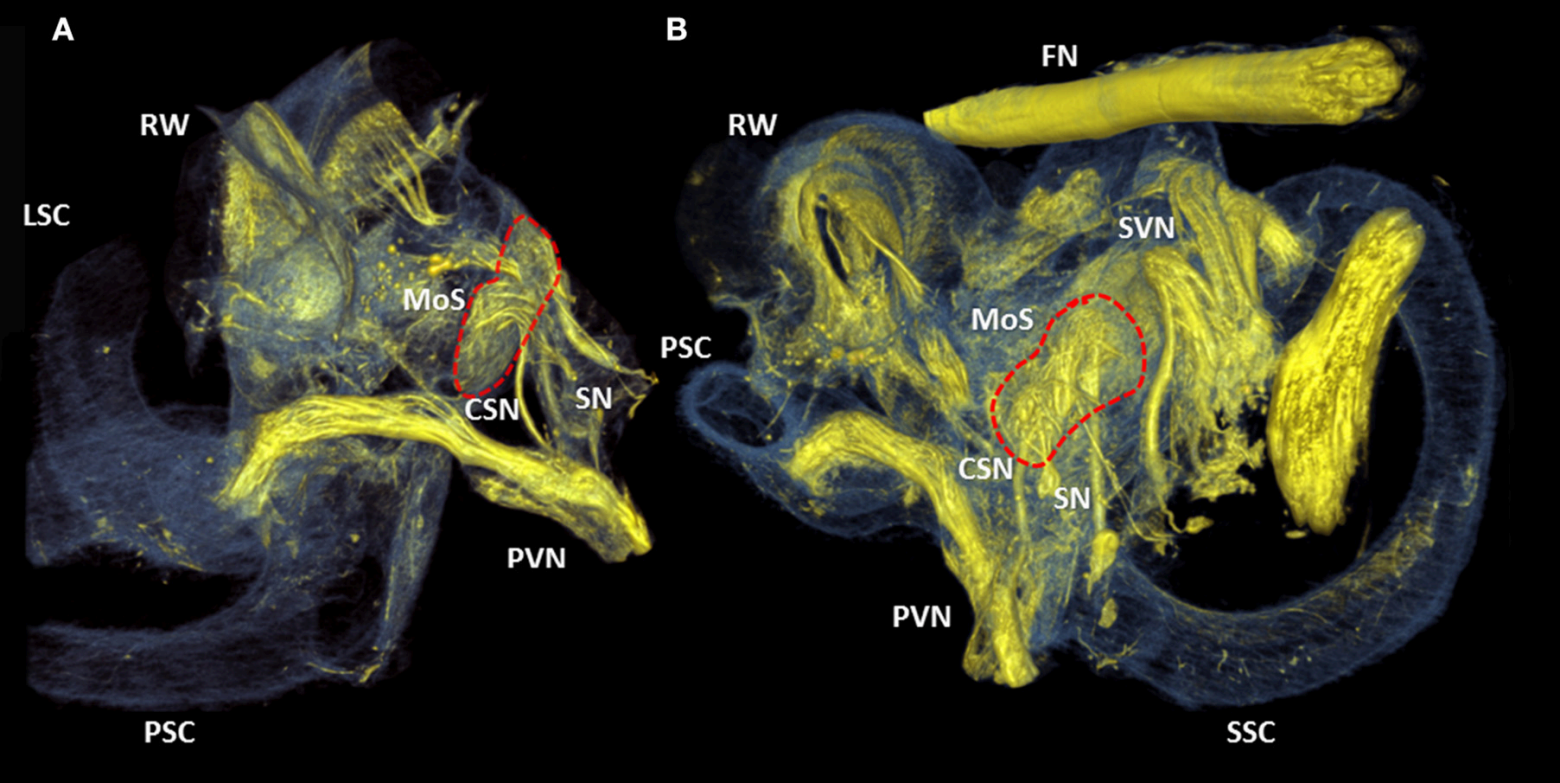
3D renderings of the cochlea and round window. Color rendering of sample S1 (5.5 μm resolution) based on intensities for nervous tissue (yellow), bone and membranous structures (blue). Two perspectives **(A,B)** of the posterior branch of the vestibular nerve. It's nerve fibers ending in the crista ampullaris of the posterior semi-circular canal. The cochleo-saccular nerve (CSN) and the saccular nerve (SN) form a neural connection with the macula of saccule (Red dashed line, MoS). RW, Round window; SVN, Superior branch of the vestibular nerve; SSC, LSC, PSC superior, lateral and posterior semicircular canal respectively.

The crista ampullaris of the posterior semi-circular canal was connected by a single fiber bundle of the posterior branch of the vestibular nerve (Figures 7A,B). A proximal divarication of the fiber bundle formed the cochlea-saccular nerve and connected the caudal part of the macula of saccule. The adjacent saccular nerve innervated the cranial part of the macula of saccule.

**Figure 8:**
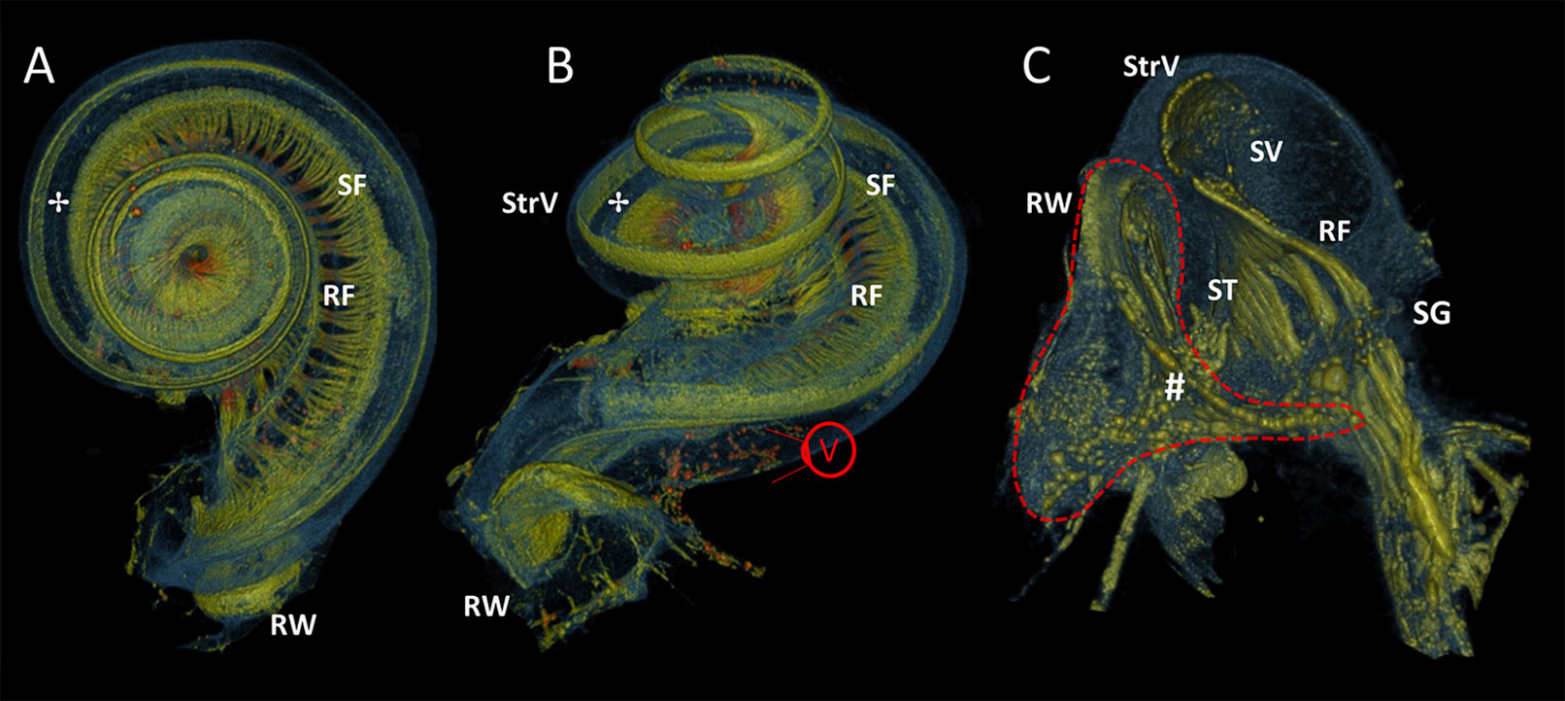
3D renderings of the cochlea and round window. Color rendering of sample S1 (5.5 μm resolution) based on intensities for nervous tissue (orange and green), bone and membranous structures (blue). Viewpoint on apex **(A)** and basal turn **(B)** of the cochlea. The radially oriented nerve fiber bundles (RF) are visually distinguishable and connect to hair cells on organ of Corti (✢).A local thickening shows the spirally oriented nerve fiber bundles (SF). RW, Round window; StrV, Stria Vascularis. **(C)** A virtually sectioned close-up from viewpoint **V**, in the direction of the basal part of the scala tympani (ST) and scala vestibule (SV). RF connect to the spiral ganglion (SG). Stained fibers (**#**) connect to the SG and may indicate neural innervation of the RW.

In Figure 8, the increased resolution (5.5 μm) exposed local intensity and morphological alterations on different positions along the radial nerve branches, indicating the presence of spiral fibers (Figures 8A,B). The round window was magnified in Figure 8C (viewpoint shown in Figure 8B). The most basal radial fibers were visible as high intensity fibers following the course of the basilar membrane at the line of demarcation between the scala vestibule and scala tympani. Fibers of high intensity connected the spiral ganglion to the round window and suggested a myelinated connection.

**Figure 9:**
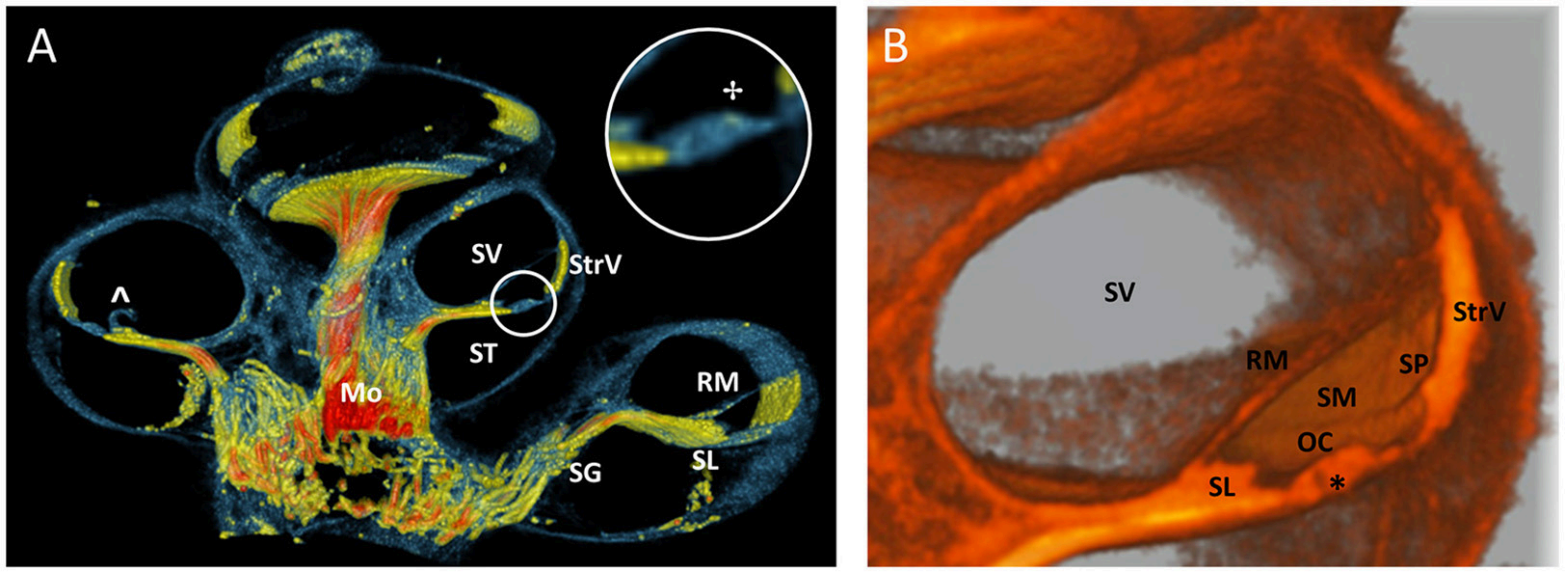
Virtual sectioned cochlea in 5.5 μm resolution. **(A)** 3D rendering of a 100 voxel thick cochlear section of sample S1 with colors identical to Figure 6. Fibers in the sectioned modiolus (Mo) are traceable toward spiral ganglion (SG) until the level of the spiral limbus (SL). The inset circle shows a magnification of the organ of Corti with an area of enhanced contrast near the outer hair cells. StrV Stria Vascularis. ST scala tympani. SV scala vestibule. RM Reissner's membrane. The RM was damaged (^∧^), probably due to preprocessing. **(B)** Monochromatic 3D rendering shows a close-up of SV and scala media (SM). Organ of Corti (OC) is virtually sectioned and reveals the tunnel of Corti (^*^). The spiral prominence (SP) is visualized as a convex bulge below the StrV.

A 100 voxel thick virtual cross sectioned rendering (Figure 9A) showed the scalae of the cochlea and thin structures such as Reissner's membrane and spiral limbus. The spirally folding nerves in the modiolus could still be appreciated because of the thickness of the cross section. An area of enhanced contrast near the region of the outer hair cells was visible in a magnification of the organ of Corti (Indicated by ✢in Figure 9A). A monochromatic close-up of a virtual cross section of the cochlea was shown in Figure 9B, where the resolving power of the 3D rendering was apparent from the level of detail of the scala media (i.e., tunnel of Corti, indicated by ^*^ in Figure 9B).

## Discussion

OsO_4_ contrast enhanced micro-CT scanning of the inner ear in combination with the presented image processing methodology made it possible to visualize relevant hard and soft tissue structures in the inner ear and was successful for all 4 samples. The algorithm for two-dimensional visualization reduced noise and increased the visibility of the micro-anatomical structures. The amount of remaining noise made this algorithm less suitable for 3D rendering. The 3D visualization algorithm enhanced the contrast of the volumes after which the designated unwanted signal was automatically removed. Overall, the quality and completeness of automatic segmentation was highest for the highest resolution (5.5 μm).

The OsO_4_ staining method yielded sufficient image contrast, as was reported by previous studies (Lareida et al., 2009; Metscher, 2009; Glueckert et al., submitted), and resulted in hyperdense neural structures such as the hyperdense cochlear nerve fibers running inside the less radiodense decalcified spongy bone in the modiolus. This differentiation could not be seen in unenhanced micro-CT images of the cochlea (Buytaert et al., 2013) and offers a complete overview of the involved structures, up to the scale of the tunnel of Corti. Due to the OsO_4_ stain, myelinated radial fibers of the cochlea could be visualized (Figures 3–5, 8, 9) until they lost their myelin at the habenular opening (Figure 9A; De Chicchis, 2002). The unmyelinated fibers were less visible. Therefore, the unmyelinated spirally oriented fibers could only be distinguished as a small accentuated thickened strip near the spiral limbus (Figures 8A,B).

The application of the described algorithms is feasible, as was evident from the reported timings and can be performed in 2D and 3D. It used software that is widely available and open source where possible. The additional manual segmentation was focussed on roughly segmenting the contour of the cochlea. The 3D rendering provided freedom to rotate volumes and to follow up on structures. Therefore, they could provide for an overview of how anatomical features relate in 3D (Figures 3, 4, 8). Specific features could be investigated selectively, as virtual cross sections or without the surrounding bony and membranous tissues (Figures 5–7, 9). Together with the spatial resolution, this facilitates future investigations and quantifications of the inner ear.

Close-ups of the basal part of the cochlea showed stained fibers with similar radiodensity as the radial fibers, passing through the spiral ganglion and connecting the round window (Figure 8C). This may indicate that there is a neural innervation of the round window as described by Rask-Andersen et al (Rask-Andersen et al., 2004). The superior vestibular ganglion gives rise to the superior branch of the vestibular nerve. This nerve can be further divided into three smaller branches, innervating the cristae of the lateral and superior semi-circular canal and the macula of the utricle. The branches that innervate the cristae of the lateral and superior semi-circular canal are called the lateral ampullary nerve and the superior ampullary nerve respectively (Lindemann, 1969). In the investigated subjects, these nerves were organized as a single strand of nerve fibers which could be subdivided in an upper (lateral ampullary nerve) and lower (superior ampullary nerve) bundle (Figure 6F). The inferior vestibular ganglion gives rise to the posterior branch of the vestibular nerve, the saccular nerve and the cochleo-saccular nerve. The latter is seen to connect to the posterior part of the macula saccule (Figures 7A,B). These findings are corresponding with studies performed on microdissections of man (Lindemann, 1969).

In the studied subjects, neural structures seemed to connect the most basal region of the cochlea to neural structures in proximity of the inferior vestibular ganglion (Figures 3A,B). These structures were indicative for the vestibulocochlear anastomosis. The vestibulocochlear anastomosis was first described at the bottom of the internal acoustic meatus by Oort (1918). The exact localisation and course differs between subjects and studies found an occurrence of 70–80% in humans (Labrousse et al., 2005; Tian et al., 2008). In 1946, experimental studies showed efferent nerve fibers in the vestibulocochlear anastomoses (Rasmussen, 1946). These fibers have their functionality in the fine regulation of sound, noise protection and adaptation, an improved signal to noise ratio and the localization of sound in a three dimensional world (Ciuman, 2010). Williams et al demonstrated that the inhibitory effects of contralateral noise on the otoacoustic emission amplitude was absent from an ear with vestibular neurectomy performed (Williams et al., 1994). Furthermore, vestibular nerve section procedures isolate dysfunctional efferent fibers, and block their efferent influence upon the cochlear. This might have an effect on tinnitus perception. A review of available literature found a mean improvement of tinnitus perception after vestibular nerve sectioning of 37.2% (Baguley et al., 2002). The visualization of these anatomical structures is interesting to identify the different varieties of the neural pathways in the inner ear and to understand the pathophysiology of otologic disorders. The mentioned structures should be investigated in a larger amount of samples to confirm these neural structures indeed match the described specifications. A larger amount of samples would also offer insights in the distribution of variations among the studied population.

### Applications

The combination of staining (Glueckert et al., submitted) and the image processing described in this manuscript could create a powerful atlas based automatic segmentation pipeline used for future inner ear studies. The described method enables researchers to visually study the micro-anatomical organization of scanned cadaveric inner ears. It opens up ways for relative quick and accurate quantifications (e.g., Organ of Corti length, neuronal distances and relations, cochlear cross-section areas or volumes) that can be applied multiple times in a non-destructive manner on the same sample. Such quantifications will allow for statistical analysis of a larger and more diverse sample of subjects. The localisation and course of neural structures and anastomoses can be compared to identify the neural variability. Visualizations of the division of the lateral and superior ampullary nerve in an upper and lower bundle (Figure 6F) are findings that potentially could be useful in the development of neural prosthetic devices. The detailed 3D view of these anatomical structures may render information about stimulation location, reach or specificity needed for electrodes to effectively transmit their information. The placement of these electrodes in, for example, vestibular implants can be simulated in detailed 3D models of varying anatomy. This may help to achieve maximal selectivity and sensitivity of electrical stimulation (Marianelli et al., 2012). Lastly, the combination of a total overview of the inner ear (Figures 3, 4) combined with selective visualizations (Figures 5–9) might also prove useful in education.

### Future perspectives

Higher resolution scans of selections of cochlear and vestibular sections could be able to visualize the smaller neural structures more selectively. The superior saccular nerve of Voit may be appreciated without the destructive process of dissection. More staining dyes are available (Metscher, 2009; Gignac et al., 2016; Glueckert et al., submitted). Research is needed to test the results of different stains (separate or combined) in temporal bones. Details up to a sub-cellular level can be visualized by high-resolution tomography of a part of an osmium stained inner ear (Lareida et al., 2009). Except for staining, histology or different imaging techniques, such as MRI (Counter et al., 2015) or transmission electron microscopy (Handschuh et al., 2013), can be combined with micro-CT images to obtain more detailed volumes. This combination could result in better visibility of, for example, the endolymphatic ducts.

## Limitations

Artifacts did exist. The hyperdense nerves introduced beam hardening artifacts in the micro-CT scans due to low peak voltage and the absence of x-ray filters. Besides beam hardening, hyperdense objects introduced blooming artifacts which may have led to overestimations of nerve thickness or underestimations of the size of hypodense object (Boas and Fleischmann, 2012; Buethe and Halliburton, 2016). Uneven distribution of the stain may have led to uneven intensity levels in parts of the micro-CT scans. Also, the membranous part of the labyrinth is susceptible to lesions and shrinkage due to preservation techniques (Glueckert et al., submitted). This may underestimate its size in living conditions.

## Conclusion

Combining high-resolution micro-CT scanning with OsO_4_ contrast staining and image processing techniques allowed for dynamic visualization and accurate assessment of micro-anatomical structures of the inner ear in 2D and 3D. Especially, the intricate folding of the neural structures of the labyrinth could be appreciated in high detail in 3D.

## Author contributions

All authors listed have made a substantial, direct and intellectual contribution to the work, and approved it for publication. MvH developed the theory and MvH and TvdB developed the methodology and performed the image processing. TvdB wrote the manuscript with support from MvH and RvdB. RG, LJ, and AS-F contributed to sample preparation and SH contributed to the CT acquisition of the samples. RvdB, NG, J-PG, HK, RG, AS-F, and AP-F supervised the findings of this work and contributed to the knowledge of anatomical structures of the inner ear.

### Conflict of interest statement

The authors declare that the research was conducted in the absence of any commercial or financial relationships that could be construed as a potential conflict of interest. The reviewer KM and handling Editor declared their shared affiliation.

## Abbreviations

CI: Cochlear implant
VI: Vestibular implant
CT: Computed tomography
Micro-CT: Micro computed tomography
MRI: Magnetic resonance imaging
X-ray: X-radiation
2D: Two-dimensional
3D: Three-dimensional
OsO4: Osmium tetroxide
PBS: Phosphate buffer saline
SRmicroCT: Synchrotron radiation micro computed tomography.
